# Indeterminate Subcutaneous Lesion of the Nasal Dorsum in an Adolescent: A Multidisciplinary Approach to a Rare Case of Spindle Cell Lipoma

**DOI:** 10.3390/dermatopathology12040040

**Published:** 2025-11-04

**Authors:** Alessandro Serrone, Chiara Rustichelli, Gian Luca Fadda, Giuseppe Riva, Massimo Rizzo, Giovanni Cavallo

**Affiliations:** 1 Department of Otorhinolaryngology, University of Turin, “San Luigi Gonzaga” Hospital, Regione Gonzole 10, Orbassano, 10043 Turin, Italy; chiararustichelli75@gmail.com (C.R.); dott.fadda@gmail.com (G.L.F.); giovanni.cavallo@unito.it (G.C.); 2 Division of Otorhinolaryngology, Department of Surgical Sciences, University of Turin, 10126 Turin, Italy; griva@cittadellasalute.to.it; 3 General Surgery Unit, Department of General Surgery, University of Turin, University Hospital “San Luigi Gonzaga”, Orbassano, 10043 Turin, Italy; m.rizzo@sanluigi.piemonte.it

**Keywords:** lipoma, rhinoplasty, adolescent, nose, spindle cell lipoma, closed rhinoseptoplasty, osteocartilaginous metaplasia, endoscopic endonasal surgery, aesthetic outcome, benign nasal tumor

## Abstract

We report the case of a 16-year-old girl presenting with a painless, clinically stable subcutaneous swelling of the nasal dorsum with a three-year history. Despite an extensive multidisciplinary diagnostic work-up—including dermatological, otorhinolaryngological, and radiological evaluations (ultrasound, CT, and MRI)—the nature of the lesion remained indeterminate. In order to achieve a definitive diagnosis while preserving the nasal profile aesthetics, the mass was entirely excised via an endoscope-assisted closed rhinoseptoplasty approach. Histopathological analysis revealed a spindle cell lipoma characterized by CD34 positivity and a Ki-67 proliferation index of less than 1%. This finding is extremely rare in terms of both anatomical location and patient age. The present case highlights the crucial role of histopathological examination in establishing the correct diagnosis, supported by a multidisciplinary assessment.

## 1. Introduction

Persistent subcutaneous nasal masses in pediatric patients represent a clinically uncommon condition and are often challenging to diagnose [1,2]. The differential diagnosis includes a broad spectrum of lesions, such as congenital anomalies—including dermoid cysts and encephaloceles—vascular malformations, and benign soft tissue tumors [3,4]. Although advanced imaging techniques, such as computed tomography (CT) and magnetic resonance imaging (MRI), have significantly improved preoperative assessment, some nasal soft tissue masses may display atypical or non-specific radiological features, making histopathological confirmation necessary for a definitive diagnosis [2,4].

In this context, spindle cell lipoma represents an exceptional finding, with few reports involving the nasal region and even fewer documented in adolescent patients. This case report describes a rare presentation and underscores the importance of close collaboration among dermatologists, otorhinolaryngologists, radiologists, and pathologists in managing ambiguous lesions with inconclusive imaging, while ensuring the preservation of facial aesthetics.

## 2. Case Report

### 2.1. Clinical Presentation

A 16-year-old girl, in good general health with no relevant comorbidities or family history, had a three-year history of a stable subcutaneous swelling on the nasal dorsum, generally asymptomatic but occasionally associated with mild erythema and minimal bloody discharge from the overlying skin.

**Figure 1:**
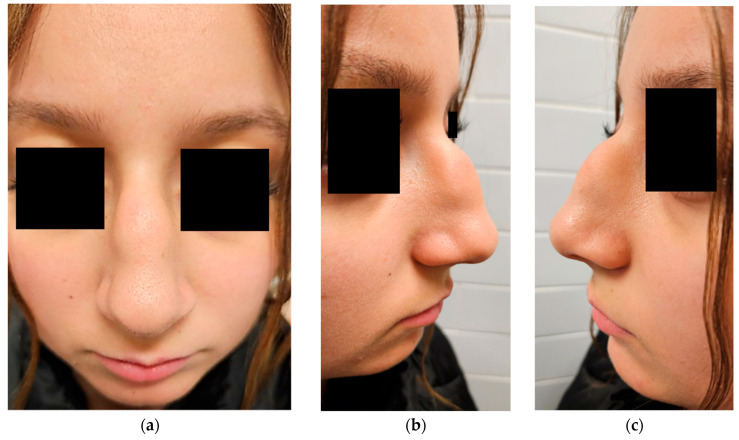
Preoperative clinical views of the nasal dorsum. (**a**) Frontal view showing a midline subcutaneous swelling; (**b**) right lateral view; (**c**) left lateral view.

On physical examination, the lesion appeared as a well-circumscribed, soft, and mobile subcutaneous mass, non-tender to palpation, with intact overlying skin and no signs of local inflammation (Figure 1). Although any potential impact on nasal symmetry could not be clearly assessed due to the overlying swelling, there were no signs of infiltration or involvement of deeper structures, nor any evidence of nasal airway obstruction.

### 2.2. Investigations

**Figure 2:**
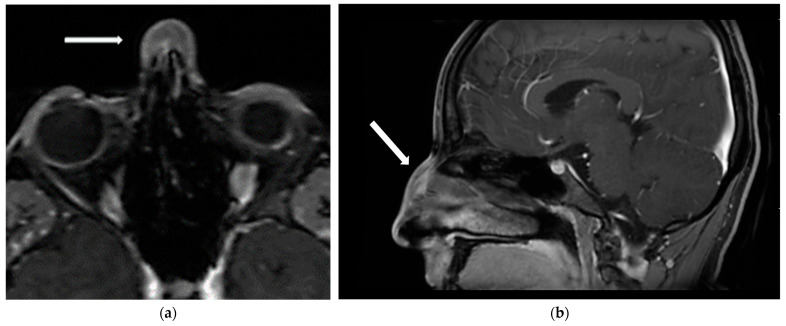
Magnetic resonance imaging (MRI) of the nasal region. (**a**) Axial T1-weighted view showing a subcutaneous lesion of the nasal dorsum (white arrow); (**b**) sagittal T1-weighted view of the same lesion (white arrow).

**Figure 3:**
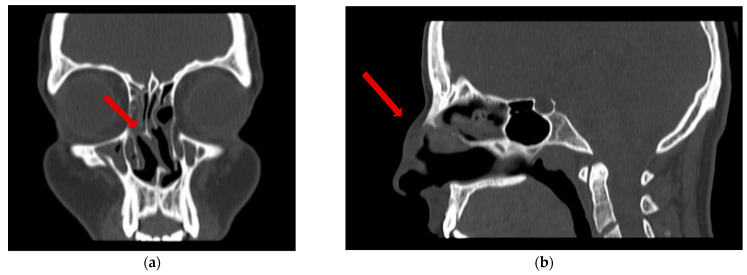
Computed tomography (CT) of the nasal region. (**a**) Coronal CT view showing sinusoidal deviation of the nasal septum (red arrow); (**b**) sagittal CT view showing subcutaneous lesion of the nasal dorsum (red arrow).

The diagnostic work-up, including ultrasound, X-ray, MRI (Figure 2), and CT (Figure 3), excluded intranasal extension, vascular malformations, and signs of malignancy but did not provide a definitive diagnosis. Flexible endoscopy confirmed the absence of intranasal masses or mucosal abnormalities, revealing only a septal deviation.

Given the persistence of the swelling and the inconclusive findings, surgical treatment was discussed in a multidisciplinary setting and deemed appropriate for both diagnostic and therapeutic purposes. Fine-needle aspiration cytology (FNAC) was proposed but declined by the patient and her parents, who preferred a single diagnostic and therapeutic procedure.

Consequently, a closed endonasal rhinoseptoplasty approach was selected, allowing complete removal of the mass without visible cutaneous scarring and with the additional benefit of correcting the septal deviation. This single, minimally invasive intervention successfully met diagnostic, therapeutic, and aesthetic-functional objectives.

### 2.3. Treatment and Outcome

**Figure 4:**
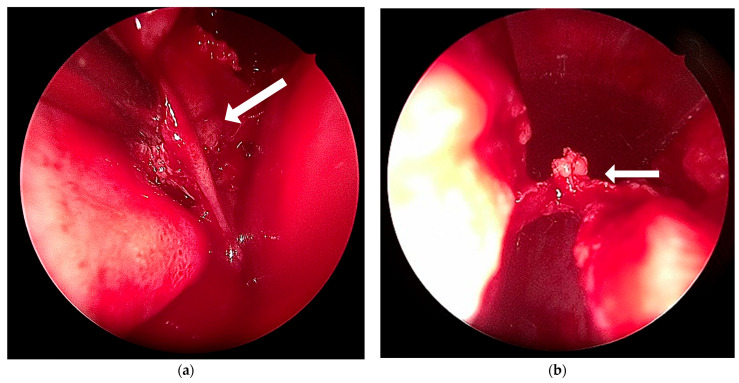
Intraoperative dissection of the nasal dorsum under 0° endoscopic visualization. (**a**) Partial exposure of the subcutaneous lesion during surgical removal (white arrow); (**b**) view of the cartilaginous nasal septum after dissection (white arrow).

The procedure was performed under general anesthesia through a closed endonasal approach with selective endoscopic assistance to ensure precise visualization. (Figure 4).

The mass was completely removed while preserving adjacent structures and maintaining the nasal contour. At the same time, the septal deviation was corrected and dorsal symmetry restored.

The postoperative course was uneventful, with no early complications, signs of infection, or bleeding. The patient was discharged on postoperative day two in good general condition.

Microscopic examination revealed a well-circumscribed but unencapsulated paucicellular proliferation of uniform spindle cells arranged in short fascicles and parallel bundles, embedded within a collagenous stroma. In some areas, focal myxoid changes were evident, blending gradually with dense collagen bundles. Scattered mature adipocytes were interspersed among the spindle cells, contributing to the heterogeneous composition of the lesion. No cytologic atypia, necrosis, or mitotic activity was observed, further supporting the benign nature of the process. A small focus of osteocartilaginous metaplasia was also identified.

**Figure 5:**
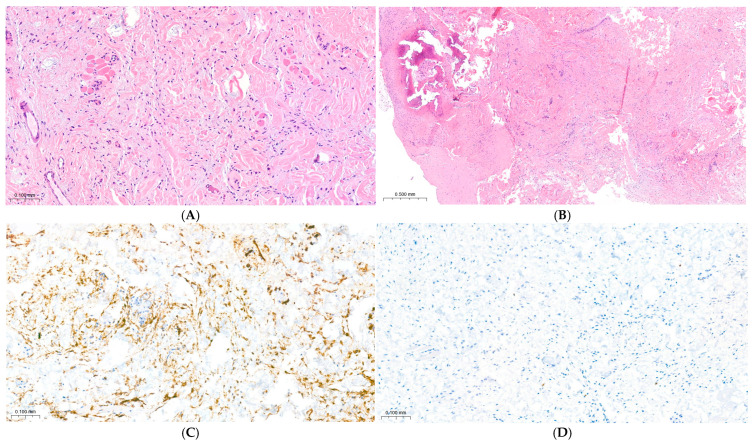
Histopathological Features of Nasal Spindle Cell Lipoma. The lesion shows the typical triad of bland spindle cells, rope-like collagen bundles, and scattered mature adipocytes. A paucicellular spindle-cell proliferation within a collagenous stroma exhibits focal myxoid change and no mitotic activity, blending seamlessly with the surrounding collagen (Panel (**A**), H&E, 20×). Focal osteocartilaginous metaplasia was also noted (Panel (**B**), H&E, 5×). Immunohistochemically, the lesion exhibited strong and diffuse CD34 positivity (Panel (**C**), IHC, 20×) and a very low proliferative index, with Ki-67 labeling in less than 1% of tumor cells (Panel (**D**), IHC, 20×).

Immunohistochemical analysis showed strong and diffuse positivity for CD34 in the spindle cell population, while the Ki-67 proliferation index was <1%, indicating extremely low proliferative activity. These features established the diagnosis of spindle cell lipoma and, in combination, effectively excluded other spindle cell neoplasms that may arise in the nasal region and mimic this entity clinically or histologically. The surgical excision margins were free of tumor (Figure 5).

**Figure 6:**
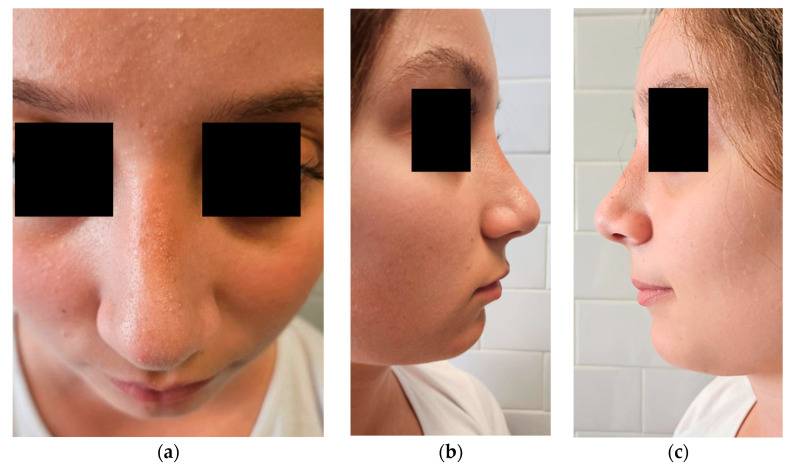
Postoperative clinical views at 3-month follow-up. (**a**) Frontal view showing restoration of nasal symmetry and absence of residual swelling; (**b**) right lateral view; (**c**) left lateral view.

Follow-up evaluations at 1 and 3 months postoperatively showed no evidence of clinical recurrence or functional or aesthetic complications. The patient expressed full satisfaction with the surgical outcome, reporting both preserved nasal function and a natural aesthetic appearance (Figure 6).

Annual clinical follow-up was scheduled for long-term monitoring.

## 3. Discussion

Spindle cell lipoma (SCL), first described by Enzinger and Harvey in 1975, is a rare benign histological variant of lipoma [5,6].

Histologically, it consists of uniform spindle-shaped cells arranged in short fascicles or sheets within a variably collagenous or myxoid stroma. The spindle cells display bland, elongated nuclei with fine chromatin and inconspicuous nucleoli, without cytologic atypia or mitotic activity. Mature adipocytes are interspersed among the collagen bundles, with their proportion varying considerably according to the morphological subtype.

Three main variants have been recognized: classic, low-fat, and fat-free. In the latter two, adipocytes are scant or absent, which may complicate diagnosis. The present case corresponds to the low-fat variant, consistent with the limited presence of mature adipocytes observed histologically. Billings and Folpe described 34 diagnostically challenging cases of this type, emphasizing the need to recognize these patterns to avoid misinterpretation as other spindle cell neoplasms [5,6,7].

Immunohistochemically, SCL shows strong and diffuse CD34 positivity, a very low proliferative index (Ki-67 < 1%), and no expression of MDM2 or CDK4. The lesion is negative for S100, which supports its differentiation from neural tumors. These histological and immunophenotypic characteristics were recently summarized in an updated review by Ohshima et al., confirming that the combination of CD34 expression, bland cytology, and absence of atypia represents the key diagnostic triad of spindle cell lipoma [6].

SCL is a slow-growing benign tumor that typically arises in the cervical, dorsal, or scapular regions of adult male patients [8]. Facial localization is extremely rare and frequently underdiagnosed. In one of the largest published series, Cheah et al. reported 33 facial cases, 27% of which involved the nose, all in adult patients [8].

In our review of the available literature, we did not identify any previous cases of SCL on the nasal dorsum in adolescent patients.

This highlights the rarity of both the anatomical location and the patient’s age group, underscoring the diagnostic and management challenges. A rare pediatric case on the hand dorsum in a 4-year-old further highlights the unusual presentation of these tumors in young patients [9].

Given the unusual nasal localization, several differential diagnoses should be considered when evaluating spindle cell lesions. The histopathological differential diagnosis of SCL includes several benign and malignant spindle cell neoplasms that may share overlapping morphological or immunophenotypic features.

The most relevant entities are atypical lipomatous tumor/well-differentiated liposarcoma (ALT/WDL), dermatofibrosarcoma protuberans (DFSP), neurofibroma, and solitary fibrous tumor (SFT). ALT/WDL may mimic SCL in spindle cell–rich areas but typically shows cytologic atypia, hyperchromatic nuclei, and amplification of the MDM2 and CDK4 genes, findings consistently absent in SCL [10,11]. DFSP consistently expresses CD34 but is distinguished by its characteristic storiform growth pattern, honeycomb infiltration of subcutaneous fat, and locally aggressive behavior [12]. Neurofibroma differs by its S-100 positivity and the presence of wavy, tapered nuclei within a variably myxoid stroma, whereas SCL lacks S-100 expression and displays rope-like collagen bundles among uniform spindle cells [13]. SFT shows a patternless architecture, stag-horn-like vessels, and immunoreactivity for STAT6, which is absent in SCL [14]. SFT may also express CD34, which represents a potential diagnostic pitfall when differentiating from SCL. Awareness of these potential diagnostic pitfalls is crucial to avoid misinterpretation and overtreatment.

This diagnostic profile is consistent with previous reviews, confirming the reliability of strong CD34 expression, low proliferative index, and absence of cytologic atypia as key features distinguishing spindle cell lipoma from other spindle cell neoplasms.

In myxoid or low-fat variants, alcian blue staining can be useful to highlight the mucopolysaccharide-rich stromal matrix, as described by Zhu et al. [7]. In the present case, however, the diagnosis was confidently established through routine hematoxylin–eosin and immunohistochemical evaluation, without the need for additional special stains.

From a clinical perspective, the differential diagnosis initially focused on subcutaneous nasal lesions presenting as localized, non-tender swellings, including dermoid and epidermoid cysts, vascular malformations, and benign mesenchymal tumors. During the initial anamnesis, the patient reported occasional episodes of erythema and secretion from the overlying skin, which were never confirmed during the clinical examination. These circumstances initially led to a suspicion of a dermoid cyst, a lesion frequently associated with inflammatory complications [2,4].

In any case, the diagnostic challenge posed by subcutaneous nasal masses prompted the use of advanced imaging techniques (ultrasound, CT, MRI) to rule out alternative possibilities, including encephaloceles and vascular malformations. Nonetheless, these investigations yielded nonspecific results and failed to demonstrate pathognomonic features or provide a preliminary diagnosis.

Due to the persistent lesion and uncertain diagnosis, complete excision was indicated. In this context, histopathological examination proved essential for establishing a definitive diagnosis and guiding therapeutic decisions [15].

Fine-needle aspiration cytology (FNAC) is a rapid and reliable diagnostic tool for cutaneous and subcutaneous swellings [16], but its interpretation should always be correlated with clinical history and, when necessary, histopathological and immunohistochemical evaluation [17].

In this adolescent patient, FNAC was proposed but declined by the patient and her parents after multidisciplinary counseling. Given the subcutaneous location, the benign radiological features, and the feasibility of achieving both diagnosis and treatment in a single surgical procedure, a direct complete excision was considered the most appropriate and conservative option, avoiding an additional invasive step in a young individual.

The closed endonasal approach enabled complete excision of the lesion while preserving nasal contour and avoiding external scars. This minimally invasive method, supported by literature showing comparable results between open and closed rhinoplasty [18], ensured both diagnostic and aesthetic adequacy. Endoscopic assistance improved visualization and precision during removal [19], and marginal excision has been reported as a safe strategy for SCL without recurrence [20].

A distinctive histopathological feature of this case was the presence of a focal area of osteocartilaginous metaplasia, an exceptionally rare finding in spindle cell lipomas and, to the best of our knowledge, not previously documented in this anatomical location.

Similar changes have occasionally been reported in conventional lipomas and are interpreted as benign reactive phenomena, secondary to fibroblastic metaplasia or local mechanical stimuli, rather than indicators of aggressive potential [12]. In our case, the metaplastic area was sharply demarcated, devoid of atypia or necrosis, confirming the benign nature of the lesion.

SCL is a tumor with an indolent clinical course and an excellent prognosis following complete excision. Recurrences are extremely rare and, when they occur, are usually associated with incomplete removal. Kubin et al. [15] reported a single recurrent nasal case, emphasizing the importance of achieving radical excision at the first intervention, whereas Zenginkinet et al. [20] observed no recurrences in their series even after marginal excisions. Similarly, Cheah et al. [8], in their series of facial cases, reported neither malignant transformation nor aggressive clinical behavior, confirming the benign nature and long-term stability of SCL.

The three-month follow-up showed complete healing, satisfactory nasal contour and symmetry, and no evidence of recurrence, consistent with the benign behavior reported in the literature [19,21,22]. This timepoint also represents a widely recognized milestone in rhinoplasty protocols for assessing wound healing, residual edema, and early aesthetic and functional outcomes, supporting the adequacy of short-term follow-up for benign lesions with low proliferative activity.

This case emphasizes that, when imaging findings are inconclusive, histopathological examination remains essential to establish a definitive diagnosis of subcutaneous nasal lesions in young patients. A multidisciplinary assessment ensures accurate identification and appropriate management.

Previous reports have described nasal spindle cell lipomas managed through different surgical routes, intended to ensure complete excision or obtaining adequate tissue for histopathological diagnosis, including external approaches [23,24]. In the present case, the complete removal achieved through a minimally invasive endonasal route confirmed the benign nature of the lesion and prevented recurrence, consistent with the findings of Kubin et al. [15].

In conclusion, surgical excision should be considered when lesions persist despite inconclusive imaging and when histopathological clarification is required to exclude malignancy. In selected low-risk cases, complete removal may replace biopsy, allowing both diagnostic confirmation and definitive management.

The integrated multidisciplinary strategy adopted in this case enabled an accurate histopathological diagnosis and resolution of the lesion in a single procedure, with no complications or recurrence at three-month follow-up.

This case emphasizes the pivotal role of histopathological examination within a multidisciplinary framework involving dermatologists, otorhinolaryngologists, radiologists, and pathologists, particularly in the conservative management of pediatric and adolescent patients with lesions in delicate anatomical areas.

## 4. Conclusions

Spindle cell lipoma of the nasal dorsum in adolescence is an exceptionally rare occurrence and represents a true diagnostic challenge due to its atypical site and non-specific radiological appearance.

When imaging is inconclusive, complete excision may represent an appropriate option to obtain adequate tissue for histopathological and immunohistochemical evaluation. In the present case, microscopic analysis revealed a well-circumscribed, low-fat variant of spindle cell lipoma showing diffuse CD34 positivity and a Ki-67 index below 1%, confirming its benign nature and excluding malignant mimics.

This case underscores the diagnostic value of detailed histopathological assessment and immunophenotypic profiling in rare spindle cell lesions of the nasal region. It further highlights the importance of multidisciplinary collaboration between dermatopathologists, radiologists, and otorhinolaryngologists to achieve diagnostic accuracy while preventing unnecessary or overly aggressive treatment.

## Data Availability

No new data were created or analyzed in this study. Data sharing is not applicable to this article.

## Abbreviations

The following abbreviations are used in this manuscript:

SCL	Spindle cell lipoma
CD34	Cluster of Differentiation 34, a transmembrane glycoprotein expressed on hematopoietic stem cells, endothelial cells, and certain soft tissue tumors
Ki-67	Nuclear protein used as a proliferation marker in histopathology
MDM2	Murine Double Minute 2
CDK4	Cyclin-Dependent Kinase 4
ALT/WDL	Atypical Lipomatous Tumor/Well-Differentiated Liposarcoma
DFSP	Dermatofibrosarcoma Protuberans
SFT	Solitary Fibrous Tumor
US	Soft tissue ultrasound
CT	Computed tomography
MRI	Magnetic resonance imaging
FNAC	Fine needle aspiration cytology
